# Abnormal grain growth mediated by fractal boundary migration at the nanoscale

**DOI:** 10.1038/s41598-018-19588-4

**Published:** 2018-01-25

**Authors:** Christian Braun, Jules M. Dake, Carl E. Krill, Rainer Birringer

**Affiliations:** 1 Department of Experimental Physics, Saarland University, Campus D2.2, 66123 Saarbrücken, Germany; 2 Institute of Micro and Nanomaterials, Ulm University, Albert-Einstein-Allee 47, 89081 Ulm, Germany

## Abstract

Modern engineered materials are composed of space-filling grains or domains separated by a network of interfaces or boundaries. Such polycrystalline microstructures have the capacity to coarsen through boundary migration. Grain growth theories account for the topology of grains and the connectivity of the boundary network in terms of the familiar Euclidian dimension and Euler’s polyhedral formula, both of which are based on integer numbers. However, we recently discovered an unusual growth mode in a nanocrystalline Pd-Au alloy, in which grains develop complex, highly convoluted surface morphologies that are best described by a fractional dimension of ∼1.2 (extracted from the perimeters of grain cross sections). This fractal value is characteristic of a variety of domain growth scenarios—including explosive percolation, watersheds of random landscapes, and the migration of domain walls in a random field of pinning centers—which suggests that fractal grain boundary migration could be a manifestation of the same universal behavior.

## Introduction

The coarsening of typical polycrystalline materials results in compact, faceted grain shapes that resemble soap bubbles. The geometric form of these grains—characterized topologically by the number of faces, edges and vertices—imparts complexity to the network of boundaries: usually, three 2D grain faces meet along each 1D edge (triple line), and four edges begin or end at each zero-dimensional vertex (quadruple point)^1^. When provided with sufficient kinetics, the network evolves in such a manner that the overall area of boundaries decreases, as this reduces the excess energy stored therein^2^. This process entails larger grains growing at the expense of their smaller neighbors, which results in the successive elimination of shrinking grains and a concomitant increase in average grain size.

Tuning the latter quantity to optimize specific properties is the task of materials processing, which exploits the response of polycrystalline microstructures to applied stresses and temperatures (thermomechanical treatment)^3^. The key challenge hereby is to promote or suppress coarsening via control of the migration of grain boundaries. Understanding boundary migration is, in turn, the basis for developing predictive models for microstructure evolution—one of the paramount goals of materials science and statistical physics^4^. In this regard much attention has been devoted to the idealized case of isotropic grain growth in two and three dimensions. Quite generally, models for the coarsening of polycrystalline microstructures presume that the excess energy of grain boundaries manifests itself in the form of a surface tension^2^, which imparts a driving force for boundary migration through the boundary’s mean curvature. When the surface tension is equal or similar in magnitude for all grain boundaries in a specimen, then the average grain size $\langle R\rangle$ grows parabolically with time (*i.e*., ${\langle R\rangle }^{2}\propto t$), and the grain size distribution evolves self-similarly, as verified by both theory^2,5^ and computer simulation^3,6,7^.

In real materials, however, the power-law nature of growth and its self-similarity can be disturbed by the presence of anisotropy (texture), inhomogeneities, the segregation of impurities at boundaries, or by second-phase particles—just to name the most prominent impediments to so-called “normal” grain growth. In such cases, coarsening can be markedly nonuniform, with a small subpopulation of grains growing rapidly to sizes more than an order of magnitude larger than those of the remaining population. Grains that grow quickly are assumed to profit from some kind of energetic and/or mobility advantage that allows their boundaries to migrate quickly through a matrix of quasi-pinned neighboring grains. This mode of growth fits the standard definition for abnormal grain growth (AGG)^3^—which is not itself a new phenomenon, having been investigated for more than 70 years^8^—and several approaches have been developed to induce or suppress its occurrence. Despite these efforts, our understanding of the mechanisms underlying AGG remains fragmented at best^9^, prompting Rollett *et al*.^10^ to call it “one of the perennially fascinating problems in materials science.”

Although the name implies a certain degree of rarity, abnormal grain growth is hardly an unusual occurrence at the micrometer grain sizes of conventional materials, and in nanoscale polycrystals AGG may be even more common than its “normal” counterpart^11,12^. Based on the latter observation, it is conceivable that nanocrystallinity could be conducive to the appearance of new modes of AGG.

## Results

**Figure 1:**
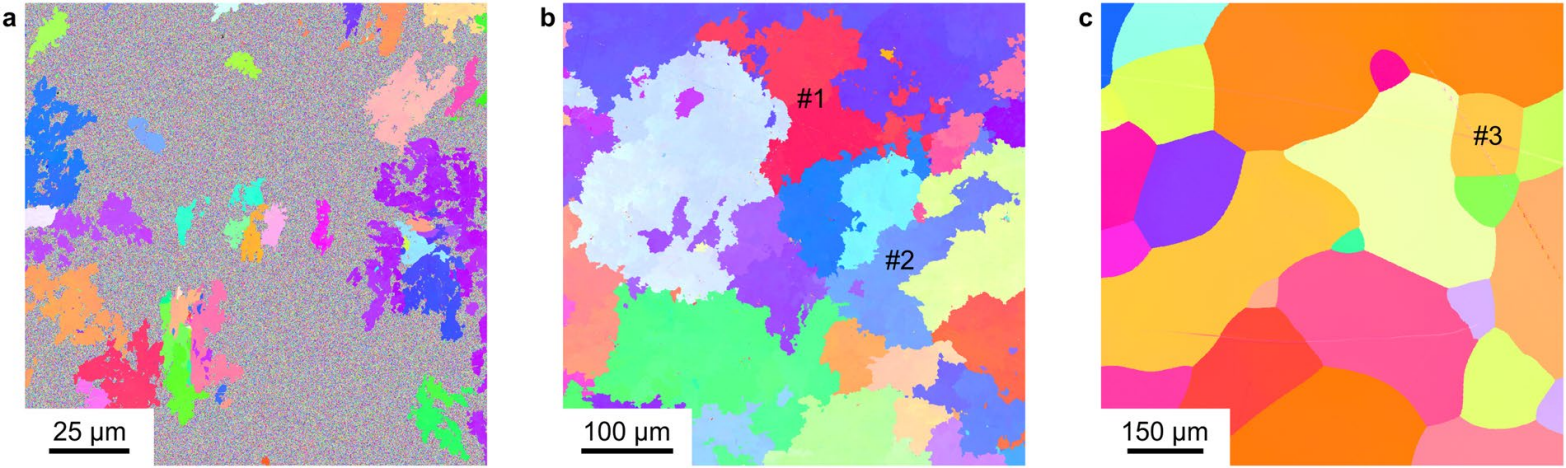
Microstructural maps of abnormal grain growth in nanocrystalline Pd-10 at% Au, recorded by electron backscatter diffraction (EBSD) following heat treatments of (**a**) 155 °C for 202 h and (**b**) 400 °C for 4 h. The speckled contrast visible between single-color micrometer-sized regions in (**a**) is caused by the presence of grains smaller than the point resolution of the EBSD technique at this magnification (0.15 *μ*m). The perimeters of abnormal grains in (**a**) and (**b**) are much rougher and more convoluted than the smooth boundaries seen in (**c**), which represents the microstructure of a Pd-10 at% Au sample prepared by arc melting and subsequent solidification.

We have investigated this possibility in Pd-based specimens having a nanocrystalline (NC) microstructure prepared by inert gas condensation^13^. In these samples, we discovered that the morphological signature of AGG can differ drastically from that encountered in conventional polycrystals: instead of maintaining approximately equiaxed shapes with smooth growth fronts (interfaces between abnormal and matrix grains), domains growing rapidly in NC Pd-10 at% Au take on rough and highly irregular morphologies (Fig. 1(a)). These abnormal grains appear to have grown by sending out offshoots in many directions, first surrounding nearby matrix grains and then consuming them, perhaps similar to the manner in which a tumor spreads into nearby tissue. During later stages of growth, the abnormally growing grains impinge upon one another, but the tortuous nature of their perimeters is preserved (Fig. 1(b)).

Closer examination of the resulting domain shapes reveals them to bear a qualitative resemblance to fractal objects^14^. Complex geometric objects, or sets, can be assigned a fractional dimension that typically exceeds the related topological dimension by a noninteger amount^15^. When a set has a fractal index, that set fills its embedding space qualitatively differently than does an ordinary geometric shape: for example, a curve with a fractal dimension significantly greater than unity has lost the usual character of a line, winding in a highly convoluted manner through two or three-dimensional space.

To assess whether an abnormal grain’s ostensible fractality withstands quantitative scrutiny, we must choose an appropriate definition for the fractal dimension of a 3D grain’s intersection with a sectioning plane. Formal definitions of fractal dimension have been derived from deterministic fractals, which are constructed by mathematical codes that permit scaling to arbitrarily small length scales^16^. A prominent example of such a definition is the box-counting fractal dimension, ${D}_{0}={\mathrm{lim}}_{\varepsilon \to 0}[\mathrm{log}\,N(\varepsilon )/\mathrm{log}(1/\varepsilon )]$, where *ε* denotes the box size and *N* the number of boxes crossed by the perimeter of the object in question^17,18^.

The box-counting dimension is particularly suited to the analysis of 2D sections of fractal objects. In order to determine *D*_0_ from such an image, the latter is overlaid with a regular square lattice of spacing *ε*, and the boxes that are intersected by the object’s perimeter are counted to give *N*(*ε*). The limit *ε*→0 is impossible to achieve in practice, being bounded from below by the underlying pixel size of the image. Consequently, the fractal dimension is estimated from the slope of a log-log plot of *N*(*ε*) versus 1/*ε*.

**Figure 2:**
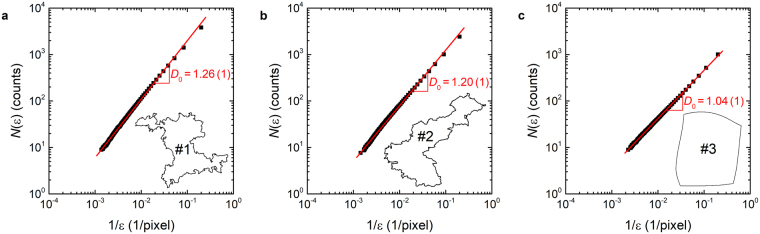
Evaluation of the box-counting fractal dimension *D*_0_, extracted from log-log plots of the number of boxes *N*(*ε*) versus the inverse box side length 1/*ε*. The red lines represent least-squares fits of straight lines to the data points (black squares), the slopes of which yield *D*_0_ values for (**a**) grain #1 and (**b**) grain #2 in Fig. 1(b) and (**c**) grain #3 in Fig. 1(c).

**Figure 3:**
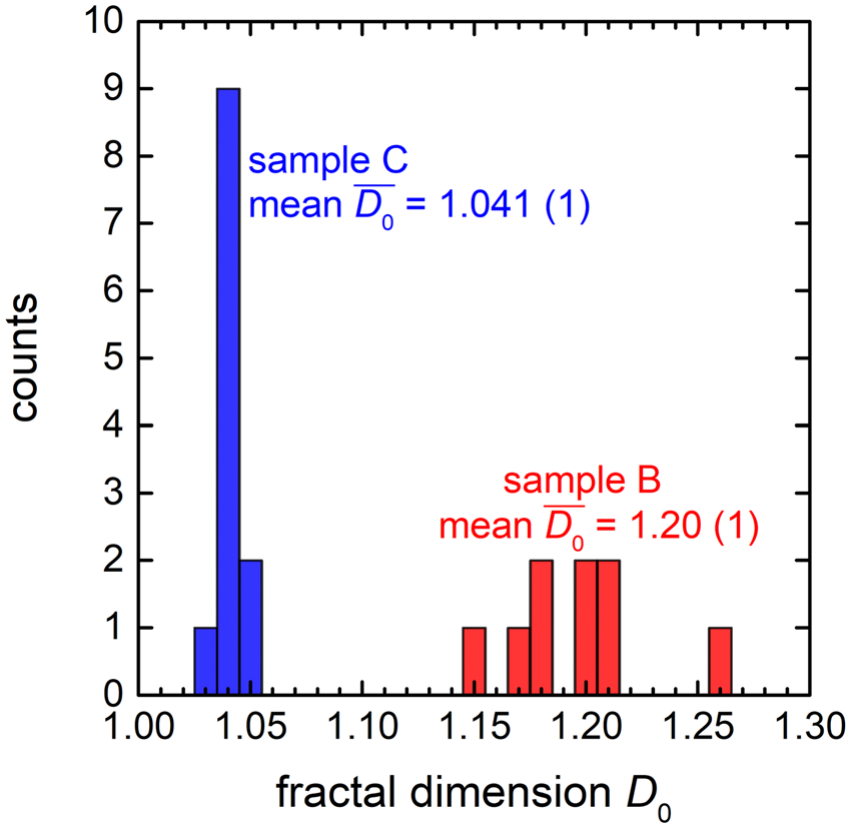
Frequency histogram of the box-counting fractal dimension *D*_0_ of the grains displayed in Fig. 1(b) and (c). The red histogram bars correspond to the fractal grain morphologies of the nanocrystalline sample following heat treatment (Fig. 1(b)), whereas the blue bars derive from the conventional polycrystalline microstructure prepared by solidification from the melt (Fig. 1(c)).

Application of this method to grains #1 and #2 in Fig. 1(b) yields the plots of Fig. 2(a) and (b). From least-squares fits of straight lines to these data points, we estimate *D*_0_ values of 1.26 ± 0.01 and 1.20 ± 0.01 for #1 and #2, respectively. We also applied the box-counting method to the microstructure of a conventional, coarse-grained Pd-10 at% Au specimen (Fig. 1(c)), extracting *D*_0_ = 1.04 ± 0.01 for grain #3 (Fig. 2(c)). At first glance, it may seem surprising that *D*_0_ exceeds unity for grain #3, but the box-counting method tends to overestimate integer-valued Euclidian dimensions by a few percent, as reflected in the values obtained for regular geometries like squares (*D*_0_ = 1.01 ± 0.01) and circles (*D*_0_ = 1.02 ± 0.01) (see Fig. S1(a) and (b) in the Supplementary Information). On the other hand, for a mathematical fractal like the Koch snowflake (fractal dimension 1.26), the same algorithm returns the true value within the error bars of the analysis (*D*_0_ = 1.26 ± 0.01, Fig. S1(c)). Fig. 3 shows the box-counting fractal dimensions of grain perimeters visible in Fig. 1(b) and (c) after excluding partially mapped grains. Red histogram bars correspond to the fractal grains of Fig. 1(b) (arithmetic mean $\overline{{D}_{0}}=1.20\pm 0.01$), and blue bars arise from the regular (*i.e*. non-fractal) conventional polycrystalline microstructure of Fig. 1(c).

## Discussion

While researching models for the development of fractal domain morphologies, we discovered a striking similarity between the grain shapes visible in Fig. 1(b) and microstructures generated by so-called “explosive percolation”^19^, the domains of which take on fractal shapes with *D*_0_ values ranging from 1.23 to 1.26, depending on the detailed nature of the site occupation rules implemented by the percolation model^19,20^. This close match to the fractal dimension of abnormal grains in NC Pd-10 at% Au suggests that percolation is a potentially fruitful framework for understanding the phenomenon of fractal AGG.

We conjecture that, in our samples, the fractal migration of grain boundaries is a manifestation of a percolation process taking place on a grid defined by the nanometer-sized matrix grains, whereby the percolation pathway is governed by details of the microstructure. In the language of percolation theory, these details can be formulated as “selection rules” that determine whether or not a neighboring site is connected to a given domain. In the case of grain boundary migration, any physically plausible selection rule will have to derive from relevant aspects of the sample microstructure, such as the misorientation relationship at grain boundaries, the distribution of segregants or impurities, the growth or dissolution of interfacial states (grain boundary complexions^21^), or the presence of obstacles—such as precipitates or pores—tending to hold boundaries in place (pinning). Although such microstructural features are common to polycrystalline materials at all characteristic grain sizes, we are aware of no previous observations of fractal grain boundary migration during grain growth, nor have we encountered a postulated connection between fractality and AGG. What, then, could be responsible for the findings reported here for NC Pd-10 at% Au?

To answer this question, we focus on the salient features of these specimens: (i) their extremely low intracrystalline defect density^22,23^, (ii) their statistically isotropic and homogeneous microstructure^23,24^, and (iii) their unusually small average grain size of ∼10 nm^25^. The first property argues against boundary migration driven by excess energy stored within grain volumes, as occurs during recrystallization. The second property implies that NC Pd-10 at% Au is devoid of macroscopic texture or deviations from a Mackenzie distribution^26^ of lattice misorientations at grain boundaries; however, such attributes have never been found to promote the appearance of fractal grain shapes in conventional polycrystalline materials. But, in conjunction with a very small grain size, statistical isotropy and homogeneity of the microstructure may establish conditions favorable to the migration of grain boundaries according to an unconventional mechanism: namely, grain rotation and coalescence^27–30^. Scaling arguments predict that, when acted upon by a torque, a grain of size *R* will rotate at a rate proportional to *R*^−*n*^, with $2\le n\le 5$^30^. Since the speed of curvature-driven boundary migration goes as *R*^−1^, there ought to be a grain size below which grain growth mediated by grain rotation is actually faster than growth by the standard curvature-based mechanism.

The driving force for grain rotation can be traced to the dependence of grain boundary energy *γ* on misorientation angle *θ*, whereby the latter quantity is a measure for the difference in crystallographic orientation of two grains meeting at a grain boundary. In the small-angle grain boundary regime ($\theta \lesssim {15}^{\circ }$), steep gradients in the *γ*(*θ*) energy landscape give rise to torques that act on the adjacent grains^31^. At random high-angle grain boundaries, on the other hand, the energy landscape is largely flat, implying that no significant torques are generated. In the small-angle region, the energy minimum at *θ* = 0° corresponds to the point at which a rotating grain would coalescence with its neighbor. Now, consider a larger grain growing into a nanocrystalline matrix: whenever the former comes into contact with a matrix grain at a small-angle grain boundary, the nanocrystallite will experience a torque driving its misorientation to zero; upon coalescence, the boundary of the larger grain advances by the spatial extent of the smaller neighbor grain, thus generating a spatial fluctuation at the length scale of the nanometer-sized matrix. As rotation-mediated growth extends further and further into the matrix—likely in the form of slender offshoots that, in turn, send out their own branches (as in Fig. 1(a))—fluctuations in the boundary morphology will be generated at all length scales greater than the size of the matrix grains. Curvature-driven migration would operate simultaneously, acting to eliminate highly misoriented grains trapped between dendritic offshoots. The resulting microstructure would be characterized by fairly compact grain shapes like those seen in Fig. 1(b), with impinged boundaries retaining the fractality induced by the underlying rotation mechanism.

Finally, it should be noted that fractal dimensions close to 1.2 have been noted for pathways and boundaries associated with a wide range of phenomena, including domain walls in strongly disordered systems (*D*_0_ = 1.2)^32^, bridge percolation (*D*_0_ = 1.22)^33^, optimal path cracks (*D*_0_ = 1.22)^34^, and watersheds of random landscapes (*D*_0_ = 1.21)^35^. These seemingly unrelated physical problems can be linked to the concept of fracturing a ranked surface^33^. In particular, watersheds have been shown to exhibit the geometrical properties of Schramm-Loewner evolution theory^36,37^, in which random curves of fractal dimension are generated from one-dimensional Brownian motion. Just as a watershed boundary is defined relative to a given surface topography, we can view rotation-mediated growth as establishing domain boundaries in reference to an energy landscape, whereby the latter has been mapped from misorientation space onto real space through the arrangement of lattice orientations that are present in the initial grain configuration. In this interpretation, the fractal dimension of grain perimeters reflects the degree of randomness of the energy landscape that underlies the mechanism for grain growth, with complete randomness corresponding to D_0_ ≈ 1.2. Moreover, just as for watersheds of landscapes having long-range spatial correlations^38^, lower values of *D*_0_ would be expected when the polycrystalline microstructure manifests a non-random misorientation distribution function, since this would introduce spatial correlations into the energy landscape driving rotation-mediated growth. It appears that growth by grain rotation and coalescence could account for the emergence of fractal grain morphologies in NC Pd_90_Au_10_, but validation of this scenario will require a tight integration of experiment, theory and simulation.

## Methods

### Sample synthesis and preparation

The nanocrystalline Pd-Au samples of Fig. 1(a) and (b)—having a gold concentration of 10 at% and an initial grain size of ∼10 nm—were synthesized by inert gas condensation and compaction^13^. Applying a compaction pressure of 1.8 GPa, we prepared discs with a diameter of 8 mm and a thickness of about 500 μm. To initiate grain growth, the annealing treatments described in the caption of Fig. 1 were carried out in a differential scanning calorimeter (TA Instruments DSC Q2000) following a heating ramp of 1 °C/min. In contrast, a reference sample of identical composition (Fig. 1(c)) was produced by fusing high-purity palladium (99.95%) and gold (99.95%) wires in an arc melter that had been evacuated 10 times to a base pressure of ∼1 mbar and purged subsequently with helium (5.0). After the arc was switched off, the melt solidified to a coarse-grained specimen having the shape of a prolate sphere. For investigations of the resulting microstructure, a section was cut from the middle of the specimen with orientation parallel to the prolate plane of the sample. For this step we used a precision saw (Buehler Isomet 1000) equipped with a diamond blade. In order to carry out a detailed analysis of grain perimeters, samples B and C were ground and polished mechanically with diamond suspensions of decreasing particle size down to 1 *μ*m. To generate a defect-free sample surface, ion beam polishing was performed until the deformation layer of the mechanical polishing was removed completely. To that end, sample B was placed in a Baltec RES 010 ion mill at 2.5 kV acceleration voltage, 2.5 mA ion current and 10° inclination angle relative to the sample surface, whereas in the case of sample C a Hitachi IM 4000 ion milling system was applied (2.5 kV accelerating voltage, 40 *μ*A ion current and 10° inclination angle). This resulted in optimally resolved Kikuchi patterns during characterization by electron backscattering diffraction (EBSD), which allowed grain shapes to be visualized based on the orientation of the underlying crystal lattice.

### Orientation imaging microscopy and determination of fractal dimension

All microscopy studies were carried out in a JEOL JSM-7000F scanning electron microscope (SEM) operated at 15 kV acceleration voltage and 5 nA beam current. The SEM was equipped with an EDAX/TSL Digiview 3 camera and EDAX/TSL OIM data collection software (version 5.2), which was used to generate the microstructural maps displayed in Fig. 1. In order to determine fractal dimensions by the box-counting method, we defined an image area of 600 *μ*m × 600 *μ*m for all micrographs, which were scanned with a hexagonal grid, a consistent step size of 0.6 *μ*m and a magnification of 160×. After setting the misorientation threshold for individual grain recognition to an angle of 5°, each fully captured grain was extracted individually from the dataset, colored in black and saved as a bitmap image (4000 × 4000 pixels) using the EDAX/TSL OIM analysis software (version 8). Finally, the grain perimeters were identified using the program ImageJ^39^, from which their fractal dimensions were determined by means of the ImageJ plugin FracLac (version 2015Sept090313a9330)^40^. Here we chose the following parameters: minimum box size 5 pixels, maximum box size 45% of the image, scaling method “Default Sampling Sizes”, and 50 grid positions. The latter parameter instructs the box-counting algorithm to count the number of boxes *N*(*ε*) intersected by the grain perimeter for 50 different selections of grid origin (for additional details, please consult the FracLac documentation). In the diagrams of Figs 2 and S1 (Supplementary Information), we plot the average value $\mathrm{log}\,N(\varepsilon )=(1/\mathrm{50)}{\sum }_{i}\,\mathrm{log}\,{N}_{i}(\varepsilon )$ against the inverse box size 1/*ε*, such that the slope of the linear regression yields the fractal dimension averaged over all grid positions. This procedure was validated against square and circular perimeters as well as against the Koch snowflake, resulting in the fractal dimension values quoted in the main text; the corresponding box-counting plots are shown in Fig. S1 of the Supplementary Information.

### Data availability

The datasets analyzed during the current study are available from C. Braun (c.braun@nano.uni-saarland.de) or R. Birringer (r.birringer@nano.uni-saarland.de) upon request.

## Electronic supplementary material

Supplementary Information
