# Supplementary material for: Abnormal grain growth mediated by fractal boundary migration at the nanoscale
Source: Sci Rep. 2018 Jan 25;8:1592. doi: 10.1038/s41598-018-19588-4 (PMC5785526; doi:10.1038/s41598-018-19588-4)
Supplement: Supplementary file 1 — Supplementary Information [file 41598_2018_19588_MOESM1_ESM.pdf]

Abnormal grain growth mediated by fractal boundary migration at the nanoscale

Christian Braun, Jules M. Dake, Carl E. Krill III, Rainer Birringer

S1. Fractal dimension of regular geometric structures

To assess the uncertainty inherent to determination of the fractal dimension D_0 by the box-counting method, the perimeters of a square, a circle and the Koch snowflake were analyzed according to the procedure described in the Methods section. Figure S1 shows a log-log plot of the number of intersected boxes $N(\varepsilon)$ vs. inverse box side length $1/\varepsilon$ for each perimeter. The fractal dimension D_0 is estimated from the slope of a least-squares fit of a straight line to the data points.

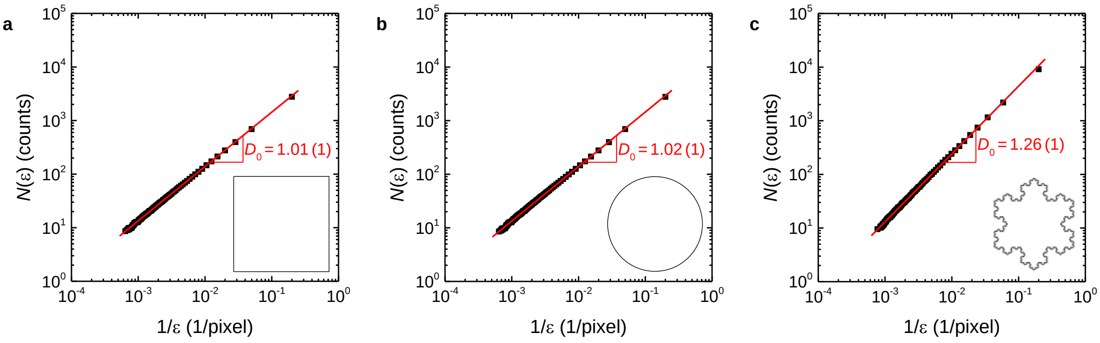

Figure S1: Evaluation of the box-counting fractal dimension D_0 from log-log plots of the number of intersected boxes $N(\varepsilon)$ versus inverse box side length $1/\varepsilon$ for the perimeters of (a) a square, (b) a circle and (c) the Koch snowflake (a mathematical fractal with fractal dimension 1.26).
